# Nonnasal extranodal natural killer/T-cell lymphoma presenting as multifocal cutaneous ulcers

**DOI:** 10.1016/j.jdcr.2023.11.028

**Published:** 2023-12-16

**Authors:** Ana Luísa Matos, Francisco Martins, Duarte Flor, Joana Xará, Carolina Afonso, José Carlos Cardoso, Joana Calvão

**Affiliations:** a Dermatology Department, Coimbra University Hospital Center, Coimbra, Portugal; b Hematology Department, Coimbra University Hospital Center, Coimbra, Portugal

*To the Editor:* Nasal-type extranodal natural killer (NK)/T-cell lymphoma is a rare subtype of T-cell lymphoma. The skin is the second most common site of involvement. As Orlowski *et al*^1^ reported, nasal-type extranodal NK/T-cell lymphoma rarely presents in an extranasal location and the diagnosis can be challenging given its variable cutaneous presentations, even mimicking inflammatory conditions.

We report a case of an 82-year-old Caucasian male presenting with a 1-month history of cutaneous ulcers and asthenia. Ulcerated lesions on the left leg and right and left forearms that were not preceded by cutaneous nodules or plaques with an undermined border were initially interpreted as pyoderma gangrenosum (Fig 1, Fig 2, Fig 3). Nonetheless, the cutaneous lesions were unusual and prompted a skin biopsy, which revealed an atypical lymphoid infiltrate throughout the dermis and subcutis, predominantly composed of medium-sized cells, with nuclear pleomorphism and karyorrhexis, exhibiting angiocentricity and adipocyte rimming (Fig 4). The infiltrate was positive for T-cell markers, with aberrant loss of CD5 and positivity for cytotoxic markers (T-cell intracellular antigen-1 and granzyme B) and CD56. *In situ* hybridization showed diffuse positivity for Epstein-Barr-encoded RNA. A computed tomography scan was negative and a positron emission tomography scan showed diffuse and heterogenous bone FDG uptake with negative bone marrow biopsy and aspiration. Blood counts were normal, except for mild lymphopenia (0.77 × 10^9^/L). Epstein-Barr viral load was 1730 UI/mL. Otorhinolaryngology excluded nasal or nasopharyngeal involvement. The diagnosis of a primary cutaneous nonnasal extranodal NK/T-cell lymphoma was established and Hematology began treatment with combination chemotherapy (modified/*mini*-*CHOP, cyclophosphamide, doxorubicin, hydrochloride, vincristine*). The dexamethasone, methotrexate, ifosfamide, L-asparaginase, and etoposide protocol, standard treatment for advanced extranodal NK/T-cell lymphoma, was contemplated but considered unsuitable given age-related comorbidities. Its main toxicities are hematological, renal, and hepatic.^2^ At 6-month follow-up, the patient is clinically well and a positron emission tomography-computed tomography showed complete remission.

**Fig 1:**
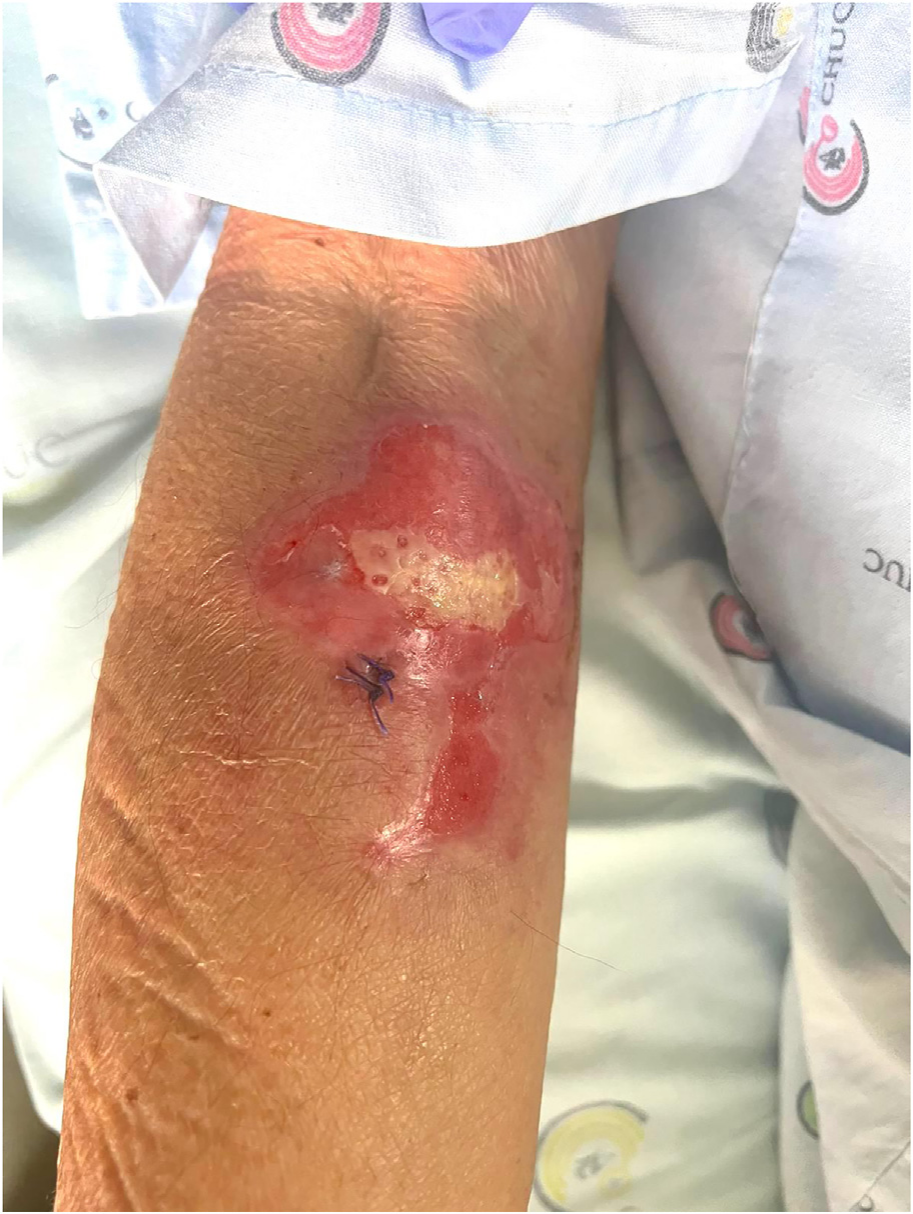
Ulcerated lesion on the right forearm.

**Fig 2:**
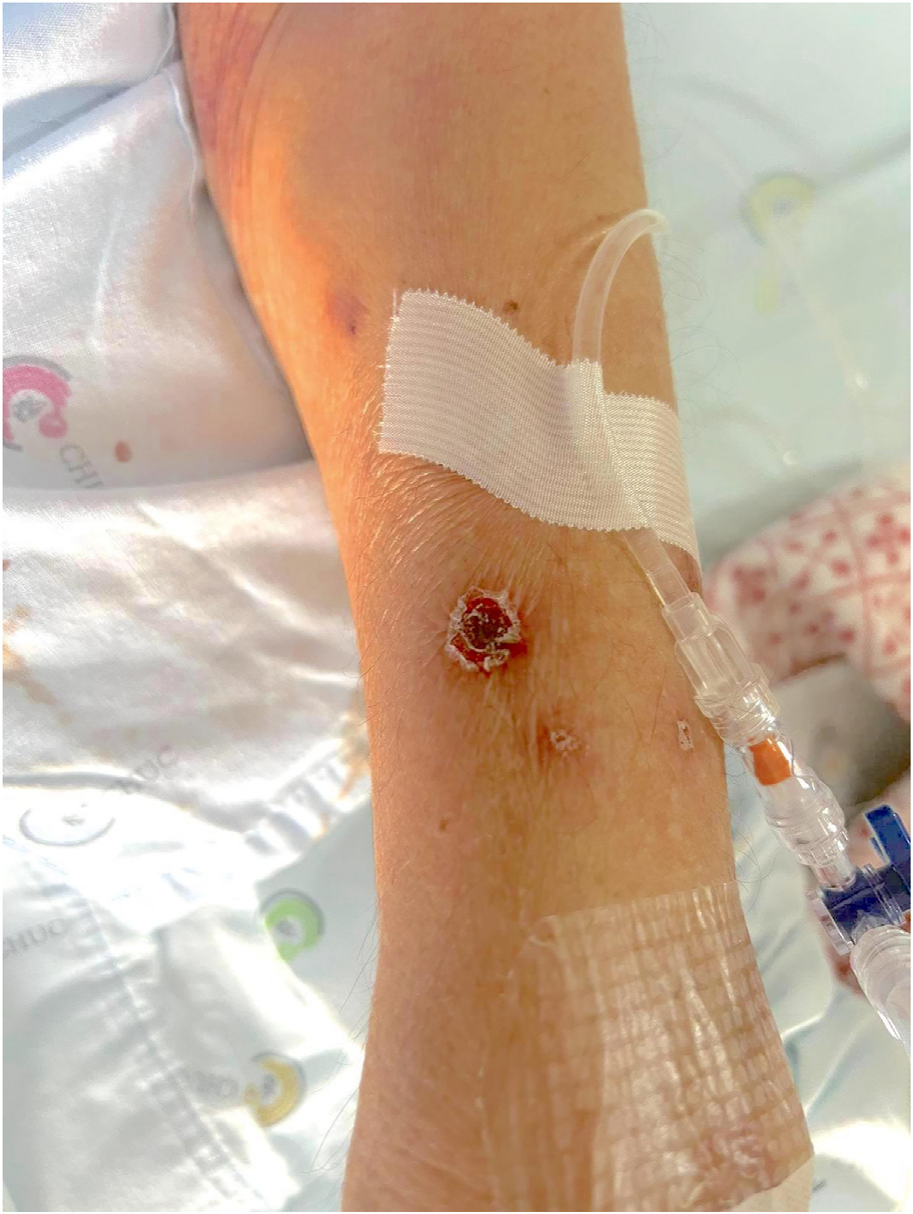
Ulcerated lesion on the left forearm.

**Fig 3:**
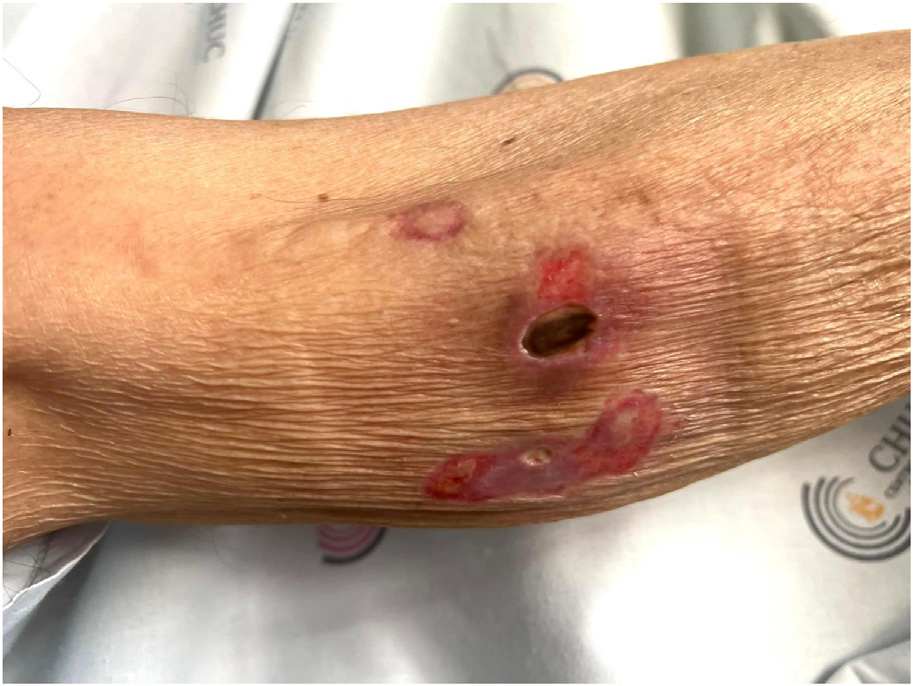
Ulcerated lesion on the left leg.

**Fig 4:**
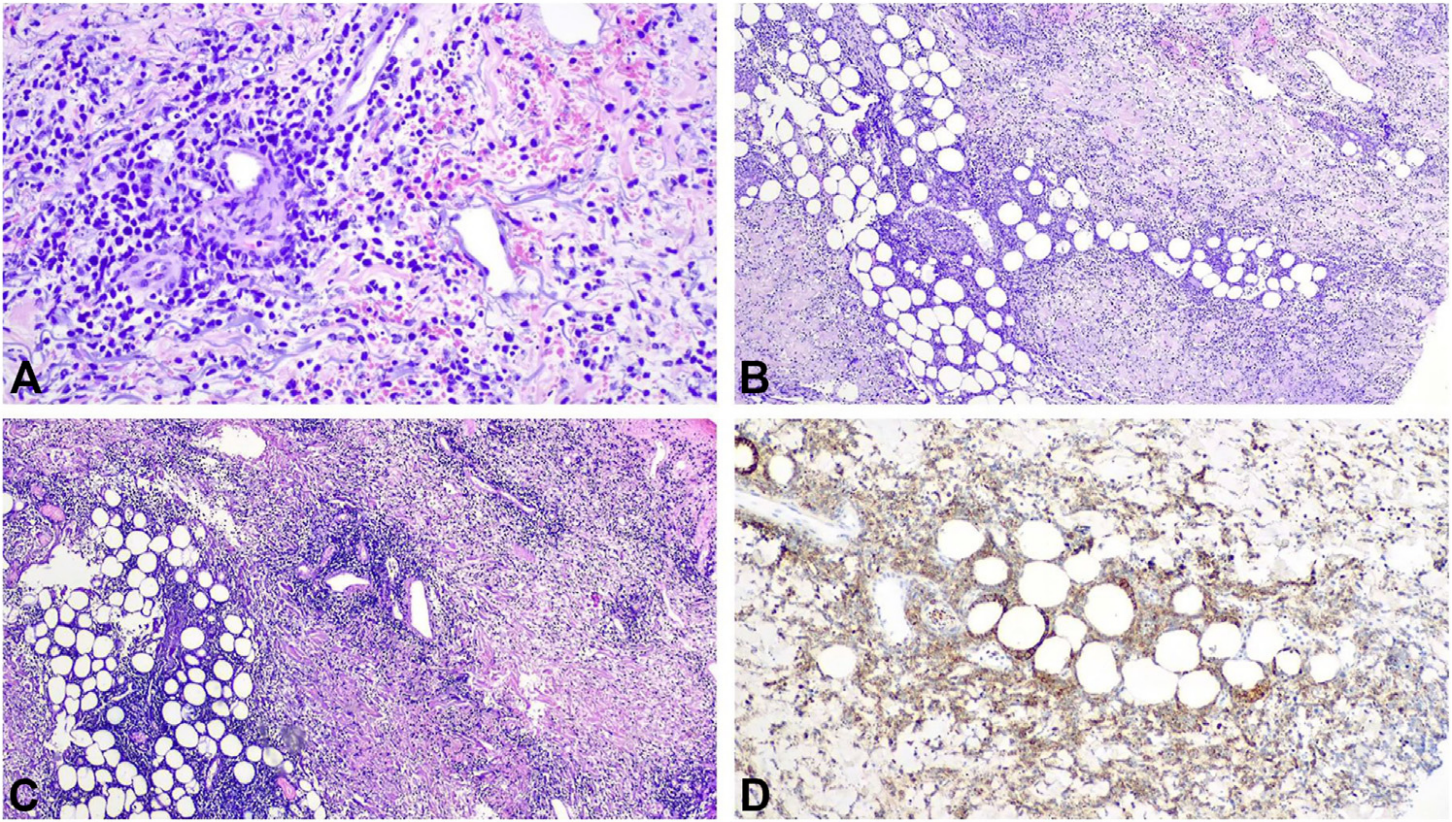
Atypical lymphoid infiltrate throughout the dermis and subcutis, with nuclear pleomorphism and karyorrhexis, angiocentricity and adipocyte rimming (**A** and **B**); diffuse positivity for Epstein-Barr-encoded RNA (**C**); positivity for CD56 (**D**).

This case illustrates the high level of clinical suspicion necessary for the diagnosis of such primary cutaneous lymphomas. Its polymorphic presentation makes clinical diagnosis extremely challenging. This, adding to the fact that these lymphomas are unusual in a nonnasal presentation, establishes this case as an uncommon and didactical one. Moreover, this case sparks the discussion regarding the staging of these lymphomas. Since Ann Arbor staging system seems to be unable to reasonably stratify the survival of nonnasal extranodal NK/T-cell lymphoma patients, Yan *et al*^3^ suggested a TNM (Tumour, Node, Metastasis) staging system in 2015. Recently, Hong *et al* proposed a new staging system suggesting that lesions confined to the nasal cavity or nasopharynx without local invasiveness and lymph node involvement should be classified as stage I, nonnasal-type disease or lesions confined to the nasal cavity or nasopharynx with local invasiveness without lymph node involvement as stage II, lesions with regional lymph node involvement as stage III and involvement of nonregional lymph node or lymph nodes on both sides of the diaphragm or disseminated disease as stage IV.^4^ This raises the discussion as to whether isolated but multifocal cutaneous disease presents with worse prognosis and should be classified as disseminated disease. Further studies and analysis of this rare entity are necessary for better understanding, staging, and treatment of these primary cutaneous lymphomas.

## Conflicts of interest

None disclosed.

## References

[1] Orlowski G.M., Tan A.J., Evan-Browning E., Scharf M.J. (2020). Primary cutaneous nasal-type NK/T-cell lymphoma presenting as purpuric nodules on the lower leg. JAAD Case Rep.

[2] Kwong Y.L., Kim W.S., Lim S.T. (2012). SMILE for natural killer/T-cell lymphoma: analysis of safety and efficacy from the Asia Lymphoma Study Group. Blood.

[3] Yan Z., Huang H.Q., Wang X.X. (2015). A TNM staging system for nasal NK/T-Cell lymphoma. PLoS One.

[4] Hong H., Li Y., Lim S.T. (2020). A proposal for a new staging system for extranodal natural killer T-cell lymphoma: a multicenter study from China and Asia Lymphoma Study Group. Leukemia.

